# Antisocial personality disorder and associated factors among incarcerated in prison in Dessie city correctional center, Dessie, Ethiopia: a cross-sectional study

**DOI:** 10.1186/s12888-022-03710-y

**Published:** 2022-01-24

**Authors:** Muhammed Seid, Tamrat Anbesaw, Shishigu Melke, Dawit Beteshe, Haydar Mussa, Amare Asmamaw, Maregu Shegaw

**Affiliations:** Department of Psychiatry, Wollo University College of Medicine and Health Science Department of Psychiatry, P.O. Box 1145, Dessie, Ethiopia

**Keywords:** Antisocial, Incarcerated, Prison, Personality, Dessie, Ethiopia

## Abstract

**Background:**

Antisocial Personality Disorder (ASPD) describes individuals who engage in repetitive aggressiveness, deceitfulness, impulsivity, and unlawful behavior. It has a broad impact on families, relationships, and social functioning, and also people with ASPD make heavy demands on the judicial system, social and mental health services. Even though ASPD is a common problem among incarcerated in prisons, it is not well studied in developing countries including Ethiopia. Therefore, this study aimed to determine the prevalence and associated factors of ASPD among incarcerated in prison in Dessie city correctional center.

**Method:**

A cross-sectional study design was conducted among 320 incarcerated in prison at Dessie correctional center, Ethiopia. The study subjects were selected by using a simple random sampling technique and the Diagnostic and Statistical Manual 5th text revision (DSM-5) was used to measure ASPD using face-to-face interviews. Social support was assessed using the Oslo social support scale (Oslo-3). The collected data were checked for completeness and entered into Epi-data Version 3.1 and then exported to SPSS version 26 for analysis. Bivariate and multivariable logistic regressions were done to identify factors related to antisocial personality disorder. In multivariable logistic regression variables with a *p*-value, less than 0.05 were considered significant and, adjusted OR (AOR) with 95% CI was used to present the strength of the association.

**Results:**

The current study showed that the prevalence of ASPD was found to be 30.6% (95% CI:25.6,35.9). In a multivariable analysis, being single [AOR = 2.33; 95%CI (1.39,3.89)], monthly income of 1000–2000 ETB (Ethiopian Birr) [AOR = 2.12; 95%CI (1.163,3.45)], reconviction [AOR = 2.37; 95%CI (1.08,5.19)], and alcohol use [AOR = 2.00; 95% CI (1.16,3.45)] were discovered to be predictors of antisocial personality disorder.

**Conclusion:**

This study revealed that nearly one-third of incarcerated in prison were found to have an anti-social personality disorder. Being single, 1000–2000 ETB income, reconviction, and alcohol users were variables that are independent predictors of ASPD. A screening and intervention program is required and further research should be needed.

## Background

By definition, a personality disorder is an enduring pattern of behavior and inner experiences that deviate significantly from the individual’s cultural standards [1]. According to the Diagnostic and Statistical Manual of Mental Disorders (DSM-5), the diagnosis of antisocial personality disorder (ASPD) is one of the common types of personality disorder, which is characterized by an enduring pattern of unlawful behavior, aggressiveness, deceitfulness, impulsivity, irresponsibility, reckless disregard for the welfare of others, and/or remorselessness manifest during adulthood [2]. Various studies have been shown that the overall prevalence rate of ASPD among incarcerated individuals is high, reaching the variations of 46 and 84% [3].

The core features of ASPD are persistent patterns of disrespect and violation of the right of others that begin in childhood or early adolescence and continue into adulthood [1]. Individuals incarcerated in prison with ASPD are charged with violent crimes such as murder, assault, armed, robbery, kidnapping, nonviolent crimes like fraud, theft, housebreaking, sex crimes like rape and indecent assault, drug offenses such as possession of or dealing in illegal substances. As a result, they are repeatedly imprisoned. It is common that up to 50% of the trial subjects are diagnosed with ASPD [4].

Due to a variety of circumstances, there is a growth in the number of inmates around the world from time to time [5]. Ethiopia’s population is incarcerated to the tune of 111,050 persons, according to 2015 global jail population figs [6].. Furthermore, in the United Kingdom, ASPD affects 63% of male remand inmates, 49% of male sentenced inmates, and 31% of female inmates [7]. As a result, people with ASPD put a lot of pressure on the judicial system, social services, mental health care, and other areas [8]. Therapy for victims of violence is expected to cost between 3 and 26% of the health budget in the United Kingdom [9]. Other studies revealed the prevalence of ASPD among incarcerated in prison were in the USA 35.3% [10], UK 62% [11], Australia 56%, [12], Nigeria 47% [13], and in Egypt 13.6% [14].

Several psychiatric disorders, notably substance abuse, anxiety disorder, and mood disorders, are highly comorbidity with ASPD. Individuals with ASPD are four times more likely to have a mood disorder, thirteen times more likely to additionally have a substance use disorder (SUD), and seven and nine times more likely to have suicidal ideation and attempt suicide, according to DSM-5. Study shows that 29% of incarcerated in prison had current mental illnesses [15]. Different factors may increase the probability of having ASPD such as age, low educational status, being male, lack of income, substance uses such as smoking cigarettes, alcohol consumptions, and marijuana uses, a history of arrest, committing crimes, other psychiatric disorders, urban residence, and deliberate self-harm [1, 13, 16–20].

The majority of the population in the prison were found in the productive age category that will be returned to their community after they complete their time at the jail. However, the emphasis given to mental health is inadequate across the general population and particularly in incarcerated in prison. This is even more in our country with limited resources and still, there is no evidence on the prevalence and associated factors of ASPD among individuals incarcerated in prison in Ethiopia, particularly in the northeast part of Ethiopia. Even though health care services for mental disorders are planned in Ethiopia, interventions against the problem have been paid less attention, which might be due to little evidence about the problem. Therefore, this study aimed to assess the prevalence of ASPD and identify the associated factors among incarcerated in prison to fill the existing gap in the literature and will help for future interventions.

## Method and materials

### Study setting and period

The study was conducted in Dessie City Correctional center, Northeast, Ethiopia from May 25–June 15/2020. Dessie city is located 401 km northeast of Addis Ababa. According to 2016–17 South Wollo Zone statistics office data, the city has 18 kebeles with a total population of 219,978 (99,822 male and 120,156 female). Concerning Dessie City Correctional Center, which is located in the South Wollo, Amhara Region, northeast, Ethiopia, has one prison which has 1250 incarcerated in prison, of which 1190 are men during the time of the study. There is only one clinic serving incarcerated in prison, with no psychiatric service.

### Study design

An institutional-based cross-sectional study design was conducted.

### Population

#### Source population

All adult individuals incarcerated in prison (18 years and above old) in Dessie city correctional center.

#### Study population

All adult individuals incarcerated in prison in Dessie City Correctional center and who were present at the time of data collection.

### Eligibility criteria

#### Inclusion criteria

All individuals incarcerated in prison aged 18 years or above.

#### Exclusion criteria

Prisoners who were seriously ill.

### Sampling procedure and sampling techniques

#### Sample size determination

In this study, the sample size was determined by using single population proportion formula. The value of “p” was taken as 50% due to the absence of a previous study that indicates the prevalence of ASPD.

$$n=\frac{{\left[z\frac{a}{2}\sqrt{p\left(1-p\right)}\right]}^2}{d^2}$$

Where d = marginal error, n = sample size, Z = calculated number from Z-table, P = point estimate = we take 50% because there is no prevalence point estimate value in Ethiopia, and other East African countries.

The sample size was calculated by using the formula for population proportion at confidence interval CI = 95%, at 95% =1.96, and marginal error = 5%.

$$n=\frac{{\left[z\frac{a}{2}\sqrt{p\left(1-p\right)}\right]}^2}{d^2}=\left(1.96\times 1.96\left(0.5\times 0.5\right)\right)\div \left(0.05\times 0.05\right)=385$$

Since our study population is less than 10,000 it is finite we can *N* = 1250

$$n=\frac{n_0}{1+\frac{n_0}{N}}=294$$

And 10% of participants were non-respondent =324 = final sample size.

### Sampling procedure

Study participants were selected using a simple random sampling method on which participants were randomly selected from the prisoner registration book by using Open Epi Random Number Generator.

### Data collection instruments

Data were collected using an interviewer-administered questionnaire, which has different subunits. Socio-demographic characteristics, criminal, psychosocial, clinical, and substance-related factors. The DSM − 5 section II ASPD Diagnostic criteria symptoms were used. The ASPD criteria symptoms; is an assessment of ASPD accordingly DSM-V which asks participants to respond either ‘Yes or ‘No’ to every 7 items, (includes [1] failure to conform to social norms, [2] repeated lying/conning, [3] impulsivity or failure to plan [4] irritability and aggressiveness, [5] reckless disregard for safety, [6] consistent irresponsibility, [7] lack of remorse), and scored on three points or above having an ASPD and cut point less than three not having an ASPD [1], thus enabling onward clinical assessment and/or intervention. The questionnaire was used to assess independent variables for the ASPDS. The internal consistency (Cronbach alpha) of ASPD in this study was 0.84. Socio-demographic factors: age, sex, employment, ethnicity, income, marital status, religion, educational status, clinical factors (family history of mental illness, prior psychiatric history), psychosocial factors: loss of loved ones, length of stay, social support, substance-related factors: (khat, alcohol, and tobacco). Oslo-3 social support scale; according to the Oslo-3 social support scale it is classified into three categories; poor support score [3–8], moderate support [9–11], and strong support [12–14, 21]. Ever use of the substance; using at least one of any specific substance for the non-medical purpose at least once in a lifetime (alcohol, khat, tobacco, cannabis, or others). Current substance use; using at least one of any specific substances for non-medical purposes for the past 3 months (alcohol, khat, tobacco, cannabis, or others). Mental illness; previously or currently diagnosed mental illness or treated in the past or currently on treatment. WHO student drug-use questionnaire was used substance use [22]. The presence of family mental illness, chronic diseases were assessed by self-report (dichotomous response of yes and no response).

### Data collection procedure

Data were collected by four BSc psychiatry professionals and one supervisor from the MSc in psychiatry holder. The study participants were asked using a written structured questionnaire through a face-to-face interview in which the data collectors were read out loud for the study participants to respond to the questionnaires. Each section of the questionnaire was prepared in English and then translated into Amharic, and to ensure its understandability and consistency, then back-translated to English. Data collectors and a supervisor have received training for 2 days on the purpose of the study, tools, how to collect data, sampling techniques, and how to handle ethical issues, including confidentiality. A pre-test was conducted (5% of the sample size) from the Kombolcha correction center before the actual data collection to identify impending problems in the proposed study such as data collection tools and to check the performance of the data collectors. The supervisor and principal investigator were given regular supervision to ensure that all the necessary data was appropriately collected. Each day throughout the data collection period, the completed questionnaires were assured for completeness and consistency. The collected data was edited and entered into the computer, then checked twice and processed appropriately.

### Data processing and analysis

The collected data were checked manually for completeness and cleaned and stored for consistency data entered in the computer using Epi-Data version 3.1, then it was exported to SPSS 26 version statistical software for analysis. Frequency, proportion, and other descriptive analyses were used. Binary logistic regression was used to determine the association between different factors and the outcome variable, and finally, all independent variables whose *p*-value < 0.25 were entered in the final model (multi logistic regression) to identify independently associated factors for ASPD. The assumption of fitness was tested by Hosmer Lemeshow’s goodness of fit test. The strength of the association was assessed using AORs with their corresponding CIs at 95%. Finally, the p-value < 0.05 was considered statistically significant.

## Results

### Socio-demographic characteristics of the study participants

A total of 320 participants were included in this study, which resulted in an overall response rate of 98.76%. Among those respondents, 281 (87.8%) were males. The mean (SD) age of respondents was 32.5 (10.5 years), with a range of 18–75 years. From all the respondents, the majority were aged between 25 and 34 years old, 146 (45.6%). The majority of the participants, 194 (60.6%), 285 (89.1%), and 168 (52.5%) were married, Amhara by ethnicity and Muslim by religion respectively. The educational status of participants showed that 117 (36.6%) of them attended grades 9–12. Regarding occupational status, 98 (30.6%) were farmers. The majority (141(44.1%) of study participants had an average monthly income greater than 2000 Ethiopian birr. A large proportion of respondents, 174 (54.4%), lived in cities (Table 1).

**Table 1:** Socio-demographic characteristics of ASPDin Dessie city correctional center, Dessie, Northeast, Ethiopia, 2020 (*N* = 320)

Variables	Category	Frequency	Percent (%)
Sex	Male	281	87.8
	Female	39	12.2
Age	18–24	62	19.4
	25–34	146	45.6
	35–44	73	22.8
	45–54	24	7.5
	55–64	9	2.8
	> = 65	6	1.9
Marital status	Single	126	39.4
	Married	194	60.6
Ethnicity	Amhara	285	89.1
	Tigray	19	5.9
	Oromo	10	3.1
	Others^a^	6	1.9
Religion	Orthodox	123	38.4
	Muslim	168	52.5
	Protestant	18	5.6
	Catholic	11	3.4
Educational status	Illiterate	57	17.8
	1–8	90	28.1
	9–12	117	36.6
	Diploma & above	56	17.5
Occupational status	Unemployed	85	26.6
	Governmental employed	56	17.5
	Non-Governmental employed	23	7.2
	Farmer	98	30.6
	Merchant	50	15.2
	Others^b^	8	2.5
Income	Less than 1199	94	29.4
	1200–2000	85	26.6
	Greater than 2000	141	44.1
Residence	Urban	174	54.4
	Rural	146	45.6

Others^a^:-Afar, ^b^:- Daily labour

### Criminal and psychosocial characteristics of the respondents

In this study, the majority 247 (77.2%) of participants had spent less than 3 years in prison. The majority, 273 (85.3%) of the participants received a guilty conviction at the time of the study. The time incarcerated at the time of interview in prison, the majority of 137 (42.8%) was 1–6 years. This study also revealed, 129 (40.3%) had been charged because of murder followed by 80 (25.0%) theft and robbery. Among the participants, 34 (10.6%) were reconvicted incarcerated in prison, their frequency of reconvicted 17 (5.3%) was twice, and 30 (9.4%) were convicted before < 5 years ago. From the total participants, majority 168 (52.5%), 56 (17.5%), and 180 (56.3%) had lost loved ones, had a history of self–harm, and had poor social support respectively (Table 2).

**Table 2:** Criminal and psychosocial characteristics of ASPDin Dessie city correctional center, Dessie, Northeast, Ethiopia, 2020 (*N* = 320)

Variables	Categories	Frequency	Percent (%)
Length of stay in prison(in a year)	Less than 3	247	77.2
	4–6	48	15.0
	7–9	13	4.1
	Greater than 9	12	3.8
Decided by the court on sentence	Yes	273	85.3
	No	47	14.7
For how long charged in prison (in the year)	1–6	137	42.8
	7–12	91	28.4
	13–18	34	10.6
	Greater than 18	14	4.4
Types of crime have been done	Murder	129	40.3
	Physical attack and try to murder	62	19.4
	Rape and abduction	25	7.8
	Theft and Robbery	80	25.0
	Others^a^	24	7.5
Convicted before the current one by the court	Yes	34	10.6
	No	286	89.4
Frequency of convicted before the current one	Once	16	5
	Twice	17	5.3
	Triple	1	0.3
For how long convicted before(in year)	< 5 year	30	9.4
	> = 5 year	4	1.3
Loss of loved	Yes	168	52.5
	No	152	47.5
History of self-harm	Yes	56	17.5
	No	264	82.5
Social support	Poor social support	180	56.3
	Moderate social support	120	37.5
	Strong social support	20	6.3

Others ^a^:- political issues, sexual assault

### Clinical and substance characteristics of the respondents

According to this finding, 44 (13.8%) of the respondents had a history of psychiatric illness. Among participants, 22 (6.9%) of respondents had a family history of mental illness, and 15 (4.7%) participants reported a history of chronic medical illness. Regarding substance use history, 62 (19.4%), 90 (28.1%), 58 (18.1%), and 38 (11.9%) were smokers, khat chewers, and cannabis users in their lifetime, respectively, as reported by the participants (Table 3).

**Table 3:** Clinical and substance characteristics of ASPD in Dessie city correctional center, Dessie, Northeast, Ethiopia, 2020 (*N* = 320)

Variables	Categories	Frequency	Percent (%)
Past psychiatric illness	Yes	44	13.8
	No	276	86.3
Family history of mental illness	Yes	22	6.9
	No	298	93.1
History of medical illness	Yes	15	4.7
	No	305	95.3
Smoking cigarette	Yes	62	19.4
	No	258	80.6
Alcohol use	Yes	90	28.1
	No	230	71.9
Khat use	Yes	58	18.1
	No	262	81.9
Cannabis use	Yes	38	11.9
	No	282	88.1

### Prevalence of ASPD in Dessie city correctional center

In this study, the prevalence of ASPDamong Dessie city correctional center was 30.6% (95%CI: 25.6,35.9).

### Factors associated with ASPD among Dessie correctional center

Bivariate and multivariable logistic regression analysis was done to identify factors associated with antisocial personality among correctional centers. On the bivariate analysis, sex, marital status, income, reconviction, history of self-harm, loss of loved ones, past psychiatric illness, family history of mental illness, smoking cigarette, alcohol use showed a *p*-value of < 0.25 and became candidates for multivariable analysis. In multivariable binary logistic regression, variables such as marital status, income, reconviction, and alcohol use were found to be statistically associated with an ASPD at a p-value less than 0.05.

The odds of ASPD among single respondents were 2.33 times higher as compared to married participants [AOR = 2.33; 95%CI (1.39,3.89)]. Those participants with a monthly income of 1000–2000 Ethio birr were 2.12 times more likely to have an ASPD as compared to participants with an income of > = 2000 [AOR = 2.12; 95%CI (1.13,3.95)]. Likewise, individuals incarcerated in prison who were convicted at least once before were 2.37 times more likely to have ASPD than individuals incarcerated in prison with no previous prison history [AOR = 2.37;95%CI (1.08,5.19)]. Furthermore, alcohol users were 2.00 times more likely than non-alcohol users to have an ASPD [AOR = 2.00; 95%CI (1.16.345)] (Table 4).

**Table 4:** Bivariate and multivariate logistic regression analysis results of ASPD in Dessie city correctional center, Dessie, Northeast, Ethiopia, 2020 (*N* = 320)

Variables	Category	Antisocial Personality Disorder		COR(95%C.I)	AOR(95%C.I)	***P***-values
		Yes	No			
Sex	Male	91(32.4%)	190(67.6%)	2.19(0.93,5.15)	2.12(0.84,5.35)	0.112
	Female	7(17.9%)	32(82.1%)	Ref	Ref	
Marital status	Single	54(42.9%)	72(57.1%)	2.55(1.57,4.16)	2.33(1.39,3.89)	**< 0.001**
	Married	44(22.7%)	150(77.3%)	Ref	Ref	
Monthly income	< 1200	33(35.1%)	61(64.9%)	1.77(0.99,3.15)	1.53(0.83,2.83)	0.171
	1200–2000	32(37.6%)	53(62.4%)	1.97(1.10,3.55)	2.12(1.13,3.95)	**0.019**
	> = 2000	33(23.4%)	108(76.6%)	Ref	Ref	
Loss of loved ones	Yes	63(37.5%)	105(62.5%)	2.01(1.23,3.27)	1.41(0.83,2.41)	0.21
	No	35(23.0%)	117(77.0%)	Ref	Ref	
History of self-harm	Yes	21(37.5%)	35(62.5%)	1.45(0.79,2.66)	0.98(0.48,2.00)	0.95
	No	77(29.2%)	187(70.8%)	Ref	Ref	
Reconvicted	Yes	17(50.0%)	17(50.0%)	2.53(1.23,5.19)	2.37(1.08,5.19)	**0.031**
	No	81(28.3%)	205(71.7%)	Ref	Ref	
Past psychiatric illness	Yes	19(43.2%)	25(56.8%)	1.89(0.98,3.63)	1.37(0.63,2.97)	0.420
	No	79(28.6%)	197(25%)	Ref	Ref	
Family history of mental illness	Yes	12(54.5%)	10(45.5%)	2.95(1.23,7.10)	2.58(0.98,6.82)	0.055
	No	86(28.9%)	212(71.1%)	Ref	Ref	
Cigarrate use	Yes	27(43.5%)	35(56.5%)	2.032(1.14,3.59)	1.06(0.50,2.23)	0.88
	No	71(27.5%)	187(72.5%)	Ref	Ref	
Alcohol use	Yes	40(44.4%)	50(55.6%)	2.3(1.42,3.95)	2.00(1.16,3.45)	**0.013**
	No	58(25.2%)	172(74.8%)	Ref	Ref	

Note: Ref: Reference

## Discussion

This study was aimed to assess the prevalence and factors associated with ASPD. The study was a first attempt to ascertain the magnitude of ASPD and its possible association with various variables among individuals incarcerated in prison in the northeast, Ethiopia. The study showed that the prevalence of ASPD was found to be 30.6% (95%CI: 25.6,35.9), which showed that a remarkable proportion of incarcerated in prison had experienced an ASPD. Regarding the relation between crimes committed and the presence of ASPD showed that, from the cause of incarceration, murder 36(27.9%), physical attack and try to murder 17(27.4%), theft and robbery 31(38.8%), rape and abduction 8(32.0%) had ASPD.

The magnitude of ASPD in this study is similar to the studies conducted in Iowa, USA (35.3%) [10], UK 25.8% [23]. Contrarily, the magnitude of ASPD in this study was lower than study results in UK 62% [11], Australia 56%, [12], Nigeria 47% [13], Spain 70.5% [24], and South Africa 46.1% [4]. The possible reason for their difference might be assessment tools, for example, ASPD was assessed using the International Personality Disorder Examination (IPE) in the UK [11], the Personality Diagnostic Questionnaire-4 in Spain, and the Mini Neuro-Psychiatric interview in South Africa were used [4], while this study used DSM-5. Also, the current study is lower magnitude than the survey reported from Nigeria’s incarcerated in prison. The possible reason might be due to tool differences and used a pre-test, post-test control group experimental design inspite of this study [13]. Furthermore, sociocultural differences,sample sizes, and demographic characteristics might be the reason for this variation.

Contrarily, the prevalence of ASPD in this study was higher than a systematic review conducted by Fazel, S & Danesh, J, which was 7.8% in western countries [25], and Egypt 13.6% [14]. This variation might be, in western older participants were included, whereas in our study most of the participants were found in adults. The prevalence of ASPD peaks in people age 24 to 44 years and drops off in people 45 to 64 years [26]. Another possible reason for the observed variation might be due to the sociocultural variation of the respondents.

In this study, concerning marital status, our study showed that those who were single are found to be more likely to have ASPD when compared to married ones. This was supported by the study conducted in Zambia [27]. Marital relationships may offer mental health benefits in terms of economic well-being and emotional and social support. The absence of family support may higher the burden associated with being single in a prison.

The current study found that monthly income 1000–2000 ETB was significantly associated with ASPD as compared to participants with income > = 2000 ETB. This is supported by another finding [1]. This is the finding that having a lower income, they have difficulty getting healthy service and care.

In this study, being reconvicted had higher significantly associated with ASPD when compared with their counterparts. This result is supported by a study carried out in Egypt [14], and the study done in Lowa, USA [10]. The reason might be due to people with ASPD frequently performing acts that result in arrest and they are unable to conform to social norms that commonly govern many aspects of a person’s adolescent and adult behavior, and their impulsion to commit a crime is genetically oriented [1]. Also, another reason might be due to ASPD is linked to violence and crime which is why people with ASPD are repeatedly sentenced and spend much of their time in imprisonment [28]. In addition, incarcerated in prison with ASPD took significantly less time to re-offend compared with those without such co-morbidity [11].

Furthermore, those alcohol users were 2 times more likely to have ASPD than non-alcohol users. This finding was in line with another finding from Lowa, USA [10], Egypt [14], University of Otego, Wellington, New Zealand [29], South Africa [4]. The reason might be due to people with ASPD have an irresponsible lifestyle and are impulsive to try new things like substances despite their consequences [30]. So the possible reason was people who drink alcohol had repeated episodes of behavioral disturbance and poor social relationships with committed criminal acts and also frequent prisoner admission.

### Limitations of the study

The study was conducted in one correctional center, the results cannot be generalized to the whole country and the study design was cross-sectional. Also, interviewer bias, recall bias, and under-reporting, denial of criminal activities, and substance use. It does not establish causes and effects. The ASPD diagnosis was based on the interview, and it lacks collateral information.

## Conclusion

In summary, this study revealed that nearly one-third of incarcerated in prison were found to have an antisocial personality disorder. Being female, monthly income, reconvicted, and alcohol user were factors significantly associated with an antisocial personality disorder. Even high proportion of ASPD reported, they did report no treatment is received in the center. This indicated that a high need for mental health awareness and services in the center.

## Data Availability

All data analyzed during this study are included in this published article. The data sets of the current study are available from [Tamrat Anbesaw], Email: tamratanbesaw@gmail.com; Mobile: + 2519–11289143, Wollo University, Dessie upon reasonable request.

## Abbreviations

AOR: Adjusted odds ratio
ASPD: Antisocial Personality Disorder
CI: Confidence interval
DSM 5: Diagnostic and Statistical Manual 5th text revision
ETB: Ethiopian birr
PCL-R: Psychopathic Checklist-Revised
PD: Personality Disorder
SPSS: Statistical Packaging for Social Science
UK: United Kingdom
USA: United States of American
WHO: World Health Organization
